# The Mechanism of Fluorine Doping for the Enhanced Lithium Storage Behavior in Cation-Disordered Cathode Oxide

**DOI:** 10.1063/4.0000795

**Published:** 2025-10-27

**Authors:** Yuanpeng Zhang

**Affiliations:** ORNL, Oak Ridge, TN, USA

## Abstract

Li-rich cation-disordered rock-salt (DRX) materials have emerged as promising candidates for high-capacity oxide cathodes. Their fluorinated variants have shown improved cycling stability with effectively suppressed oxygen loss. However, a comprehensive understanding of how fluorination impacts the multiscale structure and lithium transportation in DRX remains elusive in experiments. Herein, the neutron total scattering technique in conjunction with the advanced reverse Monte Carlo (RMC) fitting method is employed to characterize the intricate structure of Li1.16Ti0.37Ni0.37Nb0.1O2 (LTNNO) and the fluorinated Li1.2Ti0.35Ni0.35Nb0.1O1.8F0.2 (LTNNOF). Through rigorous statistical analysis, the multiscale structural evolution upon fluorination is quantified from atomic (≤5 Å) to long-range scale (≈100 Å). The local Li-rich environments around F induce a modest 2.4% increment in the number of fast Li 0TM (transition metal) channels. Crucially, at a broader scale, the proportion of 0TM channels participating in percolation increases significantly from 2.9% in LTNNO to 8.7% in LTNNOF. Fluorination improves the capacity release mainly through merging isolated fast Li channels into the percolation network. This work experimentally unravels the multiscale mechanism of fluorination-induced performance improvement in DRX materials and highlights the necessity of adopting an advanced RMC fitting method to obtain a full view of the complex structural features in developing high-capacity DRX cathodes.

